# COVID-19 Lockdown Restrictions and Online Media Consumption in Germany

**DOI:** 10.3390/ijerph18010014

**Published:** 2020-12-22

**Authors:** Tagrid Lemenager, Miriam Neissner, Anne Koopmann, Iris Reinhard, Ekaterini Georgiadou, Astrid Müller, Falk Kiefer, Thomas Hillemacher

**Affiliations:** 1 Department of Addictive Behavior and Addiction Medicine, Central Institute of Mental Health, Medical Faculty Mannheim/Heidelberg University, 68159 Mannheim, Germany; miriam.neissner@zi-mannheim.de (M.N.); anne.koopmann@zi-mannheim.de (A.K.); falk.kiefer@zi-mannheim.de (F.K.); 2. Feuerlein Center on Translational Addiction Medicine (FCTS), University of Heidelberg, 69115 Heidelberg, Germany; 3 Department of Biostatistics, Central Institute of Mental Health, Medical Faculty Mannheim/Heidelberg University, 68159 Mannheim, Germany; iris.reinhard@zi-mannheim.de; 4 Department of Psychiatry and Psychotherapy, Paracelsus Medical University, 90419 Nuremberg, Germany; Ekaterini.Georgiadou@klinikum-nuernberg.de (E.G.); thomas.hillemacher@klinikum-nuernberg.de (T.H.); 5 Department of Psychosomatic Medicine and Psychotherapy, Hannover Medical School, 30625 Hannover, Germany; Mueller.Astrid@mh-hannover.de; 6 Department of Psychiatry, Social Psychiatry and Psychotherapy, Hannover Medical School, 30625 Hannover, Germany

**Keywords:** COVID-19 pandemic, online media consumption, age and gender specific differences

## Abstract

The lockdown restrictions due to the COVID-19 pandemic have led to increased stress levels and feelings of anxiety in the general population. Problematic usage of certain online applications is one frequent way to compensate for negative feelings and stress. The current study investigated changes of online media consumption during the lockdown in Germany. Gender and age specific differences in specific online activities were assessed. *n* = 3245 subjects participated in an online survey conducted between the 8th April and the 11th May 2020. Participants’ age ranged between 18 and >55 years. A considerably high percentage (71.4%) of participants reported increased online media consumption during the lockdown. Male participants were more likely to increase their consumption of gaming and erotic platforms, while female participants reported a higher increase in the engagement in social networks, information research, and video streaming than males. The findings revealed an increased usage of all online applications during the lockdown. For the clarification whether the increase might present a risk for elevated Internet-use disorders or can be regarded as a functional and time-limited phenomenon, further studies, assessing changes in these online activities after the end of the pandemic, are needed.

## 1. Introduction

The novel coronavirus (2019-nCoV) caused an outbreak of viral pneumonia in Wuhan, China in December 2019. Due to its highly contagious nature and estimated moderate to high mutation rates, compared to the others in the category of single-stranded RNA viruses [1], the virus has been rapidly spread by travel activities across the world.

In July 2020, the Johns Hopkins University confirmed more than 17 million cases in 188 countries, more than 10 million recoveries and about 670,000 deaths [2], with the highest number of cases in the US, Brazil, and India. The tremendous increase of infected cases has prompted a large number of countries to declare restrictions as well as lockdown rules.

Thus, on 23 March 2020, the German government started to implement inevitable life-saving restrictions on social life and contacts in order to slow the spread of COVID-19 pandemic in all of the 16 partly sovereign federal countries. This so-called lockdown included closures of schools, kindergartens, and nonsystemically relevant shops (e.g., restaurants, cafes, and hairdressers), travel bans and border closures, cancellation of sporting events and various others such as concerts as well as restrictions of social contacts. These interventions led to social isolation and increased stress, anxiety, depressive symptoms, insomnia, anger, and global fear in the general population [3,4].

Moreover, the lockdown-induced social isolation might also have influenced peoples’ Internet use and, if so, this might lead to the question if the lockdown restrictions might have had an impact on an increase of prevalence rates of addictive internet use.

Since the expansion of the commercial internet in the middle of the 1990s and the observation that the extensive use of some online applications shows strong similarities to substance related addictive behavior (e.g., loss of control, psychological withdrawal symptoms, persistent desire or unsuccessful efforts to stop, tolerance, etc.), a keen scientific interest on the clinical relevance of problematic online media usage has been demonstrated. Most studies assessing underlying psychological and neurobiological mechanisms of internet use disorders have been published on Internet gaming [5] followed by social networking [6,7], and online pornography [8,9]. Based on these findings, indicating that the etiological factors of behavioral addictions are comparable with substance related disorders, Brand et al. developed an explanatory model for the initiation and maintenance of addictive behavior (i.e., the Interaction of Person-Affect-Cognition-Execution (I-PACE) model [10,11]). In particular, studies on gaming disorders showed a high clinical relevance. This led to the inclusion of ‘gaming disorder’ as a diagnosis under the classification of ‘Disorders due to substance use or addictive behaviors’ in the 11th edition of the ICD (ICD-11; World Health Organization, 2019).

A recent online survey, assessing the addictive potential of different types of online media consumption revealed, among other things, significant positive correlations between Internet addiction symptoms (measured with the Internet Addiction Test, IAT; [12]) and online surfing, use of auction websites, as well as streaming videos.

These results might indicate that apart from online gaming, social media usage, and online pornography, also other types of online behavior might be associated with a higher risk for developing an Internet-use disorder and should be considered more deeply into research. Video streaming services, such as Netflix, YouTube, or Amazon prime are considered as one of the most popular Internet applications [13]. Recent study findings indicate excessive binge watching (defined as watching > 2 episodes on one sitting of a series) to be accompanied by addiction-related negative consequences, such as loss of control, craving, social problems, as well as neglect of duties [14,15,16]. Furthermore, impulsivity [17] and depressive symptoms [16,18,19] have been shown to be associated with binge watching. A recent study of Löber et al. (2020) revealed independent pathways to contribute to certain negative consequences of binge watching. Thus, depressive symptoms showed to be a significant predictor for craving and social problems. Impulsivity was significantly associated with loss of control over binge watching. Furthermore, depressive symptoms as well as impulsivity were both related to the consequence of neglecting duties [16]. Studies on information research and its addictive potential have not been published so far.

It has recently been reported that the gaming industry seems to be one of the economic sectors that benefitted the most from the COVID-19 lockdown. Although gaming can be regarded for the majority as an enjoyable activity of everyday life, it can also increase the risks to develop an addictive behavior if certain environmental and psychological factors are present. In particular, male young adults show a high risk of developing a gaming disorder [12,20,21]. Based on the I-PACE Model of Brand et al. (2019) [10,11], which assumes that the development of addictive behavior is influenced by interactions between neurobiological and psychological factors (e.g., impulsivity and depression) as well as motivational variables (e.g., the relief of a negative mood) and learning aspects (e.g., reward expectancies), it can be hypothesized that lockdown-induced social isolation might be associated with depressive symptoms and an increased online media consumption.

One study, investigating online activities of adolescents (aged 10–17 years) during the lockdown in Germany reported an increase of 75% in the daily time spent in gaming [9]. Additionally, a comparable effect was observed for social media usage. However, there was no comparison to older participants. The findings of a recent online survey on 6416 participants in China indicated 46.8% of the subjects reported increased dependence on internet use during the COVID-19 lockdown, 16.6% of the participants rated the hours of internet use longer [22]. The authors estimated an increase of the prevalence rate of severe internet dependence of about 23% during the lockdown. Lockdown related increase of the usage of specific digital applications such as gaming, social media, streaming, information, and erotic platforms as well as gender and age specific differences in the usage have not previously be considered.

To address the question, an online survey was conducted in Germany to investigate changes in the usage of different digital applications during the lockdown. In accordance to previous findings, indicating an elevated risk for the development of gaming disorder in young male adults [12,20,21] and its relation to coping with negative feelings, we additionally assess whether, in particular, young male participants displayed an increase of gaming during the lockdown. To our knowledge, this is the first study assessing media usage on specific applications during the COVID-19 lockdown restrictions.

## 2. Materials and Methods 

### 2.1. Recruitment of Participants

Data of *n*= 3245 participants (*n*_female_ = 2074) with an age range between 18 and >55 years were collected online via SoSci Survey Version 2.5.00-i (SoSci Survey GmbH, Munich, Germany) from 8th April to 11th May 2020. Apart from the assessment of changes in online media usage during the lockdown, participants’ alcohol drinking behavior, tobacco usage [23], and gambling behavior as well as their eating and buying behavior were investigated. The survey was promoted via print and social media channels as well as radio interviews. Participants aged between 18 and 80 years were invited to participate in the survey. Before taking part in this online survey, all participants were informed about the procedures involved and provided written informed consent in accordance with the Declaration of Helsinki and the EU General Data Protection Regulation. The cover page of the survey included information on what the survey is about, the reason for conducting it, how the data will be used, and the anonymous nature of the survey. The study protocol was approved by the ethics committee of the University of Heidelberg (registration number: 2020-552N). Table 1 gives an overview of demographic data of the sample.

### 2.2. Assessment Instrument

Besides sociodemographic variables (eight items), the survey included 20 questions asking participants about their online media consumption (i.e., gaming, pornography, social media, information research, and streaming) as well as alcohol, tobacco [24], food, gambling, and shopping before and during the lockdown. It was developed at the Department of Psychiatry and Psychotherapy of the University Hospital Nuremberg and the Department of Addictive Behavior and Addiction Therapy at the Central Institute of Mental Health Mannheim, Germany. For the assessment of participant’s consumption of different online applications before and during the lockdown, two separate questions were posed (“How often did you use the following applications for private purposes” (question 1) “before the lockdown restrictions”; (question 2) “during the lockdown restrictions”). The answers were given on a 4-point Likert scale (1: never, 2: rarely, 3: often, 4: very often). Apart from online pornography sites, each application in one question involved the following examples (1) gaming (e.g., online role playing games, strategy games, other browser or fun games, etc.) (2) social media (e.g., Facebook, Instagram, Twitter, etc.), streaming (e.g., YouTube, Netflix, etc.), information research (e.g., Wikipedia, online-news, etc.). Besides, all participants had to rate the extent of their perceived stress due to COVID-19 pandemic on a scale ranging from 0 = not at all to 11 = yes, very much.

### 2.3. Data Analyses

Statistical analyses were conducted using the IBM SPSS statistical package, version 24.0 (IBM Corporation, Armonk, NY, USA). In a first step, we evaluated the subjective general increase in online media usage during the lockdown based on a self-report question (“Did your media consumption change since the beginning of the COVID-19 lockdown restrictions?”).

Next the percentage of participants who increased, decreased, or showed a consistently high or low consumption of the five different online activities (gaming, pornography, social media, information research, streaming) was assessed. For this purpose, crosstabs, based on the dichotomized variables of consumption before and during the lockdown (0 = aggregating responses ‘never’ and ‘rarely’; 1 = aggregating responses ‘often’ and ‘very often’) were created.

In order to assess significant changes in the consumption during the lockdown, we conducted paired samples *t*-tests based on the items, on which participants had to rate their consumption frequency before and during the lockdown. The responses were given on a 4-point Likert scale, ranging from ‘never’ to ‘very often’. We used parametric procedures for analyzing change variables in order to benefit from the flexibility of the models and the easier interpretability of the results. Thereby we took advantage of the robustness of the tests due to our large sample size and the central limit theorem.

In the next step, ANOVAs were conducted to investigate significant main effects of the factors age (four categories) and gender on changes of consumption in all investigated applications (dependent variables: differences between responses on the consumption before and during the lockdown). Due to the low number of subjects (*n* = 9) reporting to belong to the diverse gender category, we excluded this subgroup out of these analyses. Therefore, only male and female subjects were compared to each other. Additionally, according to our hypothesis that in particular male young adults show a higher increase in gaming activities during the lockdown, the ANOVA specific interaction effects of gender and age on gaming consumption were assessed. The significance level for all tests was set at *α* = 0.050.

In a final step, linear regression analyses were performed in order to investigate the influence of participants perceived stress levels due to the pandemic lockdown on changes in online media consumption.

## 3. Results

### 3.1. Descriptive Statistics

**Figure 1:**
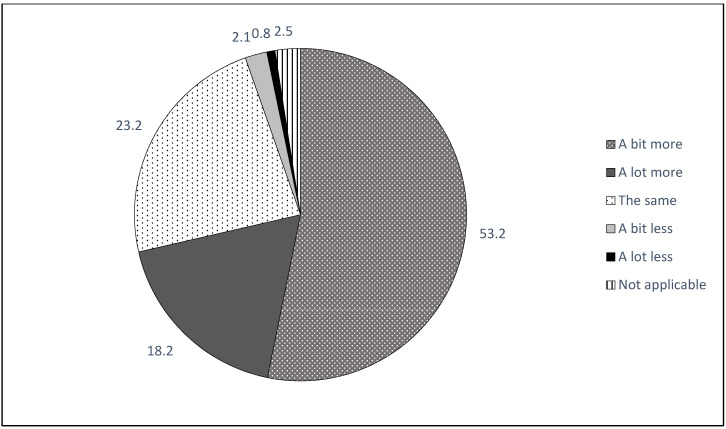
Descriptive self-reported change in online media consumption during the COVID-19 lockdown.

Descriptive results of participants’ estimation of changes in their online media consumption (question: “Did your media consumption change since the beginning of the COVID-19 lockdown restrictions?”) are displayed in Figure 1. The majority of participants reported an increase in their online activities, with 53.2% using online media a bit more and 18.2% using it a lot more than before.

### 3.2. The Consumption of Specific Online Media Applications

Table 2 shows the percentage of participants who displayed an increased, a decreased, or a constantly high or low consumption on the five specific online applications.

The results revealed that most participants did not change their consumption behavior during the lockdown. The highest percentage of users of social media, information research, and streaming reported a consistently high usage. In contrast, users of gaming and online pornography showed the highest percentage of consistently low usage.

For subjects who changed their behavior, there were higher percentages of participants who increased instead of decreased their usage during the lockdown in all applications. The highest percentage of increased usage was observed for streaming (15.9%) and information platforms (13.7%), followed by social media (9.3%), gaming (9.2%), and erotic sites (4.1%).

In a third step, we tested whether the self-reported consumption of specific online activities had significantly changed during the lockdown. Table 3 shows the results of paired sample *t*-tests. The findings revealed significantly increased use of all applications. The effect sizes ranged from low (in gaming and the use of erotic platforms) to approximately medium (in the use of social media, information, and streaming).

### 3.3. Differences in Increased Usage of Specific Applications between Age Categories and Gender and Its Interaction

In the next step, differences in the increased usage in each application before and during the restrictions among age categories and gender were tested. Analyses of variance (dependent variable: differences of responses between the self-reported usage before and during the lockdown) revealed significant main effects of the factors age group and gender on all investigated applications. As shown in Table 4, males showed a significantly higher increase in online gaming and usage of online erotic services than females. Females reported a higher increase in the consumption of social media, information research, and streaming than males. Participants aged between 18 and 24 years showed the significantly highest increase in gaming and usage of erotic platforms compared to all other age groups. The increase of streaming and social media use was significantly lower in the oldest age group (more than 55 years) compared to the other groups.

Participants belonging to the age groups from 25 to 54 years showed a significantly higher increase in information research compared to the oldest age group (>55 years).

In a final step, according to previous findings, indicating an elevated risk for the development of gaming disorder in young male adults, we fitted a model with main effects and interaction for gaming. Here, a significant interaction effect between age group and gender (F = 6.89; *p* < *0*.001; η2 = 0.006) was found. It indicates that males aged between 18 and 24 years showed the highest increase in gaming.

To clarify whether the interaction effect was influenced by the participant’s working condition during the lockdown we included a dichotomous variable ‘working in a system relevant job during the pandemic lockdown’ as an additional factor in the analyses. The results revealed no significant main effect of this factor on the increase in all investigated applications. Besides, no interaction effects between age group, gender, and working condition on gaming were found. Thus, the findings indicate that the gaming-increase in young males did not significantly change as a function of the working condition during the lockdown.

### 3.4. Effects of Pandemic-Related Stress on the Changes in Usage of Specific Online Applications

As demonstrated in Table 5, linear regression analyses revealed perceived stress during the lockdown restriction (0 = not at all, 11 = yes, very much) to be a significant predictor for increased online activities. However, the explained variance was low, ranging between 0.5% and 3%.

## 4. Discussion

The aim of the current study was to assess changes in the usage of online media consumption during the COVID-19 lockdown in Germany.

The descriptive analyses of this online survey revealed that 71.4% of the participants estimated a general increase of their online media consumption during the lockdown. However, after comparing the responses of self-rated frequencies on specific online activities before and during the lockdown, most participants reported no changes in their online behavior during the lockdown. Even if every participant rated his use as increased on only one specific application, a total of 52.2% would show elevated online activities during the pandemic lockdown. This is a high discrepancy to the general estimation of increased online usage (71.4%), assessed by one item. In particular, users of gaming and online pornography reported the highest percentage of consistently low usage (73.2% and 86.7%). One reason for this discrepancy might be the tendency to underestimate the time spent online, as one specific characteristic for problematic online behavior. A recent review [25] stated that one reason for this underestimation of time during gaming might be the strong attentional focus required by the game. Concerning online pornography consumption, the high percentage of consistently low reported usage might have been caused by feelings of shame [26]. Another reason could be that our survey did not cover the whole range of possible internet activities that might have increased during the pandemic, e.g., remote work meetings, online schooling/teaching, listening to podcasts, online shopping.

Despite the high number of participants reporting a consistent usage, also a considerable part of participants rated their frequency of online gaming, social networking, streaming, information research, and the use of erotic sites as increased during the COVID-19 lockdown. Significant increases of the usage for all applications were also detected by means of paired sample *t*-tests. The effect sizes ranged from low (in gaming and the use of erotic platforms) to approximately medium (in the use of social media, information, and streaming). Thus, the highest increase was observed in the use of streaming (16%) and information platforms (14%), followed by social media (9%), gaming (9%), and erotic sites (4%).

As mentioned above, Ioannidis, Treder [12] observed significant, albeit low, correlations between these different online activities and scores of the Internet addiction test, indicating also a certain risk for the development of addictive behaviors. However, the question whether addictive usage of these types of online activities has been increased since the lockdown should be investigated in further studies. The current findings implicate that healthcare professionals should currently be highly vigilant towards an increased Internet consumption of their clients and should furthermore inform them about the risks of developing an addictive behavior. Additionally, preventive campaigns might be helpful to support people in finding more functional coping mechanisms.

Regarding the gender specific increase of usage during the lockdown, females showed an increase of their consumption in particular in information research, social networking, and streaming compared to the male participants. Males reported a significantly higher increase in online erotic and gaming activities. Furthermore, we observed that the younger participants also showed a generally higher increase in gaming, streaming, and pornography during the lockdown than older persons. However, it should be kept in mind that these effects might be confounded with the fact that the younger age group might be less likely to have a fulltime job, which could facilitate their engagement in these online activities. This aspect is supported by our data, revealing 65.7% of the youngest age category to be school or university students (47.7%) or trainees (18.0%).

Our findings are in line with previous study findings, indicating that men are generally more often engaged in online gaming [12,20,21] as well as in the usage of erotic platforms [12,23] than women. Women instead use social media more often than men [27].

In the current study, we observed that this effect also holds true for the increase in consumption during the lockdown. This indicates that male and female participants increase their habitually preferred activities rather than engaging in new applications.

We further observed that in particular male participants of the youngest age group increased their online gaming behavior during the lockdown. According to previous research [12,20,21] indicating a higher risk for the development of addictive gaming in this subgroup, our results raise the question, if the lockdown presents a vulnerable risk factor for problematic online gaming consumption.

A recent study concerning gaming behavior of adolescents during the COVID-19 pandemic indicated an increased usage for people who experienced that gaming relieves stress [28].

This finding is in line with previous research showing that problematic gaming and usage of social media is often used as a coping technique to handle negative emotions, a phenomenon which is often described as escapism [29,30].

Accordingly, our data revealed a significant association between pandemic-related stress and the increase of usage in all online activities during the lockdown. However, the determination coefficients, explaining the variance of pandemic related stress on the specific online activities, were very small. It should be kept in mind that we did not assess anxiety disorder or depressive symptoms that might have been even more strongly induced than stress by the social isolation during the lockdown.

Apart from the interpretation that the increased consumption of online activities during the COVID-19 lockdown might elevate the risk for problematic usage, it can be also assumed that the use of those applications that involved social aspects were increased to cope with social isolation in a functional way. These online activities (i.e., social networking, gaming, and streaming) might have also helped people to reduce feelings of loneliness, and to stay in contact with friends and relatives during the contact restrictions. Therefore, the increased usage of these applications might have been a normal and functional reaction during the lockdown and should not only be regarded as generally risky. Thus, it might have been interesting to longitudinally investigate if participants with increased online media usage during the lockdown were able to decrease their consumption again after this period.

Some limitations of the study have to be addressed. The sample was recruited exclusively online and might not be representative of the general population due to a selection bias. Hence, people who take part in online surveys might have a higher Internet affinity than the general population. However, the sample size was quite large which raises the credibility of the findings. It is also not possible to deduce any causal conclusions of our results due to the cross-sectional nature of the study. Therefore, longitudinal studies should have been conducted. Besides, the study did not consider dysfunctional online media use before and during the lockdown. Therefore, a data-based assumption that problematic use of gaming or other applications has increased due to corona related restrictions cannot be given. A further limitation is that changes in online media consumption were only assessed by only two items assessing the usage of each application before and during the lockdown.

## 5. Conclusions

In conclusion, apart from a high percentage of participants who did not change their online media usage, our study findings revealed a considerable part of participants that reported an increase of online gaming, social networking, streaming, information research, and the use of erotic sites during the COVID-19 lockdown. Gender and age-related differences indicated a higher increase of social networking, information research, and streaming activities in female participants. Males reported a significantly higher use of online erotic and gaming activities. Furthermore, younger male subjects showed elevated online gaming. To our knowledge, this is the first study assessing online behavior during the pandemic restrictions and, therefore, the data should be regarded as a first description.

On the one side, the findings of increased online media consumption during the lockdown might indicate an elevated risk for the development of addictive behavioral disorders. On the other side, the consumption could also be regarded as a functional coping strategy in order to feel socially connected during the lockdown situation. For the clarification of whether the increase of online activities might have an impact on the incidence of Internet-use disorders or can be regarded as a functional and time-limited phenomenon, further studies assessing changes in these online activities after the end of the pandemic are needed.

## Figures and Tables

**Table 1:** Demographic data of the sample.

Variables	*n* = 3245	
	*n*	%
*Age*		
18–24 years old	394	12.1
25–34 years old	880	27.1
35–54 years old	1317	40.6
>55 years old	654	20.1
*Gender*		
Female	2074	63.9
Male	1162	35.8
Diverse	9	0.3
*Living situation*		
Alone	794	24.5
With partner	1118	34.5
With children	135	4.2
With partner and children	703	21.7
With parents	211	6.5
Other forms	284	8.8
*School years*		
<11 years	1010	31.4
11< x ≤ 13 years	755	23.5
>13 years	1452	45.1
*Working in a system-relevant job*		
Yes	1325	41.9
No	1839	58.1
*Employment before the lockdown*		
Full-time	1706	52.7
Part-time	741	22.9
School/university/in training	365	11.3
Pension	238	7.3
Jobless	40	1.2
Housewife/-man	66	2
Other	84	2.6
*Changes in employment during the lockdown*		
Yes	1309	45.6
No	1560	54.4

**Table 2:** Percentages of participants’ different behavior patterns before and during the lockdown.

	Consistently High Usage (Often/Very Often before and during the Lockdown)	Consistently Low Usage (Never/Rarely before and during the Lockdown)	Increased Usage (Never/Rarely before and Often/Very Often during the Lockdown)	Decreased Usage (Often/Very Often before and Never/Rarely during the Lockdown)
Gaming	15.7	73.2	9.2	1.9
Erotic	7.8	86.7	4.1	1.4
Social media	64.4	25.0	9.3	1.4
Information research	70.5	13.0	13.7	2.8
Streaming	50.8	31.5	15.9	1.8

Note. High usage: often or very often before and during the lockdown; Low usage: rarely or never before and during the lockdown; Increase: rarely or never before and often or very often during the lockdown; Decrease: often or very often before and rarely or never during the lockdown.

**Table 3:** Results of the paired samples *t*-tests assessing differences of the online media consumption before and during the COVID-19 lockdown restrictions.

Application	M_diff_	SD	SE Mean	t (3244)	*p*	d
Online games	0.11	0.51	0.01	12.30	<0.001	0.22
Online erotic services	0.03	0.41	0.01	4.66	<0.001	0.08
Social media	0.27	0.57	0.01	27.06	<0.001	0.47
Online information research	0.32	0.65	0.01	27.77	<0.001	0.48
Video/streaming services	0.33	0.67	0.01	28.27	<0.001	0.49

Note. Significant increases are printed in bold. M_diff_ = Mean difference.

**Table 4:** Main effects of age groups and gender on changes in pre–post online media consumption.

	Age Group											Gender						
	18–24		25–34		35–54		>55		F (3, 3228)	*p*	η^2^	Male		Female		F (1, 3228)	*p*	η^2^
	M	SD	M	SD	M	SD	M	SD				M	SD	M	SD			
Gaming	0.31 ^a^	0.69	0.15 ^b^	0.55	0.068 ^c^	0.46	0.03 ^c^	0.32	33.00	<0.001	0.032	0.14	0.55	0.10	0.48	4.98	0.026	0.004
Erotic	0.15 ^a^	0.56	0.02 ^b^	0.42	0.02 ^b^	0.37	0.02 ^b^	0.31	12.02	<0.001	0.012	0.07	0.494	0.01	0.335	17.55	<0.001	0.005
Social media	0.33 ^a,b^	0.62	0.35 ^a^	0.57	0.26 ^b^	0.58	0.16 ^c^	0.49	15.99	<0.001	0.013	0.21	0.53	0.31	0.59	18.92	<0.001	0.003
Information research	0.30 ^a,b^	0.66	0.37 ^a^	0.68	0.34 ^a^	0.65	0.23 ^b^	0.60	4.78	0.003	0.004	0.19	0.59	0.39	0.67	70.00	<0.001	0.014
Streaming	0.42 ^a^	0.73	0.37 ^a^	0.65	0.33 ^a^	0.68	0.21 ^b^	0.59	10.25	<0.001	0.009	0.28	0.59	0.36	0. 70	10.24	0.001	0.003

Note. Dependent variable: The mean differences of responses (1: never, 2: rarely, 3: often, 4: very often) of self-reported consumption before and during the lockdown (range: −3 to +3). Different superscript letters indicate significant pairwise post-hoc differences between age categories by Tukey HSD. a: 18–24 years; b: 25–34 years; c: 35–54.

**Table 5:** Regression coefficients regarding pandemic related stress levels and changes in online media consumption.

	B	SE	Beta	t (1, 3216)	*p*-Value	R^2^
Gaming	0.011	0.003	0.073	4.153	<0.001	0.005
Erotic	0.012	0.002	0.094	5.358	<0.001	0.009
Social media	0.030	0.003	0.175	10.092	<0.001	0.031
Information research	0.035	0.003	0.175	10.072	<0.001	0.031
Streaming	0.036	0.003	0.181	10.410	<0.001	0.033
